# OptiBIRTH: a cluster randomised trial of a complex intervention to increase vaginal birth after caesarean section

**DOI:** 10.1186/s12884-020-2829-y

**Published:** 2020-03-06

**Authors:** Mike Clarke, Declan Devane, Mechthild M. Gross, Sandra Morano, Ingela Lundgren, Marlene Sinclair, Koen Putman, Beverley Beech, Katri Vehviläinen-Julkunen, Marianne Nieuwenhuijze, Hugh Wiseman, Valerie Smith, Deirdre Daly, Gerard Savage, John Newell, Andrew Simpkin, Susanne Grylka-Baeschlin, Patricia Healy, Jane Nicoletti, Joan Lalor, Margaret Carroll, Evelien van Limbeek, Christina Nilsson, Janine Stockdale, Maaike Fobelets, Cecily Begley

**Affiliations:** 1 Queen’s University Belfast, Belfast, Northern Ireland UK; 2 National University of Ireland Galway, Galway, Ireland; 3 Hannover Medical School, Hannover, Germany; 4 Universtià degli Studi di Genova, Genoa, Italy; 5 University of Gothenburg, Gothenburg, Sweden; 6 Ulster University, Jordanstown, Belfast, Northern Ireland UK; 7 Department of Public Health, Interuniversity Centre for Health Economics Research (I-CHER), Vrije Universiteit Brussel, Brussels, Belgium; 8 Association for Improvements in the Maternity Services, Surrey, UK; 9 University of Eastern Finland, Kuopio University Hospital, Kuopio, Finland; 10 Academie Verloskunde Maastricht, Maastricht, the Netherlands; 11 Entando, Belfast, Northern Ireland UK; 12 Trinity College Dublin, Dublin, Ireland

## Abstract

**Background:**

Despite evidence supporting the safety of vaginal birth after caesarean section (VBAC), rates are low in many countries.

**Methods:**

OptiBIRTH investigated the effects of a woman-centred intervention designed to increase VBAC rates through an unblinded cluster randomised trial in 15 maternity units with VBAC rates < 35% in Germany, Ireland and Italy. Sites were matched in pairs or triplets based on annual birth numbers and VBAC rate, and randomised, 1:1 or 2:1, intervention versus control, following trial registration. The intervention involved evidence-based education of clinicians and women with one previous caesarean section (CS), appointment of opinion leaders, audit/peer review, and joint discussions by women and clinicians. Control sites provided usual care. Primary outcome was annual hospital-level VBAC rates before the trial (2012) versus final year of the trial (2016). Between April 2014 and October 2015, 2002 women were recruited (intervention 1195, control 807), with mode-of-birth data available for 1940 women.

**Results:**

The OptiBIRTH intervention was feasible and safe across hospital settings in three countries. There was no statistically significant difference in the change in the proportion of women having a VBAC between intervention sites (25.6% in 2012 to 25.1% in 2016) and control sites (18.3 to 22.3%) (odds ratio adjusted for differences between intervention and control groups (2012) and for homogeneity in VBAC rates at sites in the countries: 0.87, 95% CI: 0.67, 1.14, *p* = 0.32 based on 5674 women (2012) and 5284 (2016) with outcome data. Among recruited women with birth data, 4/1147 perinatal deaths > 24 weeks gestation occurred in the intervention group (0.34%) and 4/782 in the control group (0.51%), and two uterine ruptures (one per group), a rate of 1:1000.

**Conclusions:**

Changing clinical practice takes time. As elective repeat CS is the most common reason for CS in multiparous women, interventions that are feasible and safe and that have been shown to lead to decreasing repeat CS, should be promoted. Continued research to refine the best way of promoting VBAC is essential. This may best be done using an implementation science approach that can modify evidence-based interventions in response to changing clinical circumstances.

**Trial registration:**

The OptiBIRTH trial was registered on 3/4/2013. Trial registration number ISRCTN10612254.

## Background

Rates of caesarean sections (CSs) are increasing, yet decreases in maternal and perinatal mortality are not seen when more than 10% of births in a population are by CS [1]. Between 1990 and 2014, average CS rates increased from 6.7 to 19**.**1% globally, and from 11**.**2 to 25% across Europe [2]. In 2010, CS rates in 31 countries and regions in Europe ranged from 14**.**8% in Iceland to 52**.**2% in Cyprus [3] with highest rates among women who had had a previous CS [3]. These were between 45 and 55% in the Netherlands, Norway, Finland and Iceland, 64% in Germany, approximately 90% in Italy, and not reported in several countries, including Ireland. Similarly, CS rates in Australia ranged from 11**.**8 to 47**.**4% across 81 hospitals [4] with rates of 82**.**1% among 61,894 maternities with a previous CS [5]. Less than half (49%) of the variation in repeat CS rates in the latter study was explained by differences in women’s characteristics and hospital practices, indicating the association of other, non-clinical, factors [5]. A recent systematic review by the World Health Organization shows that 106 out of 169 countries have CS rates greater than the 9 to 16% level above which no decrease in maternal or neonatal mortality is seen [6]. CS rates exceed 40% in some countries, including the Dominican Republic (59%), Brazil (56%), Egypt (63%), and Turkey (53.1%) [7]. Given the short and long-term adverse effects of CS, these rates cause concern [8].

Elective repeat CS (ERCS) and planned vaginal birth after CS (VBAC) for women with a prior CS are both associated with benefits and harms [9]. Most studies report an increase in adverse maternal and neonatal outcomes following repeat CS [10–12]. The findings of one systematic review reported that maternal mortality is decreased by planned VBAC compared with ERCS, with nine fewer deaths per 100,000 women [13]. Significant increases in perinatal mortality were seen for “trial of labour” (0**.**13% compared with 0**.**05% for ERCS), but the authors concluded that, as the absolute risk of perinatal death is low, the evidence suggested that VBAC was a reasonable choice for the majority of women [13].

In response to rising CS rates in the United States, the National Institutes for Health and the American College of Obstetricians and Gynaecologists (ACOG) issued guidelines recommending “trial of labour after CS”, as they termed it, in 2010 [14, 15]. Two years later, 44% of hospitals in California still did not permit planned VBAC, and one tenth of the 56% of hospitals that did allow planned VBAC reported that fewer than 3% of women who had had a previous CS had a vaginal birth [16]. This indicates how difficult it can be, and how long it can take, to change clinicians’ opinion and by doing so, impact on women’s self-determined preferences to have a VBAC.

Findings from a recent systematic review of clinician-focussed interventions showed that the only strategy that significantly increased VBAC rates was an educational intervention provided by an opinion leader [17]. A study of 44 clinicians practising in three countries with high VBAC rates (> 45%) [18], and a similar study based on 71 clinicians from countries with low VBAC rates [19] showed comparable findings related to trust in the clinician-woman relationship, a positive attitude of all centrally involved, early follow-up and fear reduction. However, differences shown related to the decision-making process, parameters for VBAC, organisational support and resources, and clinical expertise, which indicate different attitudes and views among clinicians in countries with high and low VBAC rates.

Information sessions and decision-aids for women during pregnancy did not increase VBAC but did significantly decrease their decisional conflict and increase their knowledge of mode of birth [20]. Therefore, the promotion of VBAC requires the provision of evidence-based information to women on the benefits and limitations of VBAC [21] and the options open to them [22]. The need to provide better information for women and to improve VBAC rates led to the development of the OptiBIRTH intervention, which has been compared with usual care for women with one previous CS in this cluster randomised trial in Germany, Ireland and Italy [23].

### Registration

The OptiBIRTH trial was prospectively registered in the ISRCTN Registry before randomisation of the clusters (maternity units) to intervention or control (ISRCTN10612254), and the protocol was published [23].

## Methods

### Study design

A cluster randomised trial of 15 small, medium and large maternity units (annual births of 1800 to 8500) in both urban and rural locations in Germany, Ireland and Italy, with VBAC rates of 35% or less. Five maternity units took part in each country, with the 2 units in Hannover (Germany) being allocated to the same group to avoid having an intervention and a control site in the same city. A sixth German maternity unit was withdrawn from the trial following recruitment of only three participants during the pilot phase. The pilot study was conducted in 2014 from January to April (Germany), February to March (Ireland) and February to April (Italy). Minimal changes were made to data collection, selection, enrolment processes and the length of the clinician information session during the pilot phase but, as planned, the main trial results (presented in this paper) exclude women recruited during the pilot study.

### Randomisation and blinding

The unit of randomisation was the maternity unit (the “site”). Primary analyses considered women’s data at the level of each site as a whole, rather than at the level of women recruited. This was decided in order to investigate the effects of the OptiBIRTH intervention on a cultural change in practice, affecting the care of all women with one previous CS at the site, regardless of whether or not they were recruited to the trial. Random allocation of sites to intervention or control was carried out in advance of approaching women to participate. The maternity units that had agreed to join the trial were matched by their annual number of births and then by VBAC rate in each country, in either pairs or triplets. They were then randomised 1:1 or 2:1 to intervention or control, respectively [23]. This resulted in six randomised comparisons. Blinding of clinicians and participating women was not possible because they were part of the intervention. However, the trial team were blinded to the results of the trial until recruitment was closed and the data analyses completed.

### Eligibility

Pregnant women at a participating site who were: aged 18 years of age or over at time of booking; had one previous lower segment CS (not a classical/high vertical incision); spoke the language of the country where they were recruited (German, English or Italian); and gave written informed consent to participate.

Pregnant women with known multiple pregnancy at time of booking were ineligible. (As noted in the results, one woman was recruited and subsequently found to be pregnant with twins.)

### Interventions

Sites randomised to the intervention group received a complex, innovative programme of evidence-based antenatal strategies, developed following three systematic reviews [17, 20, 21], and four qualitative studies with clinicians and women in both high and low VBAC countries [18, 19, 24, 25]. Technology-assisted learning resources were also developed using motivational systems and instructional design theory [26]. The intervention involved the provision of evidence-based education for women (two antenatal classes), clinicians (one-hour session), access to optional online educational resources and mobile/computer applications, introduction of communities of practice (women and clinicians sharing knowledge), appointment of midwife and obstetric opinion leaders, audit and peer review of CS rates in each site, and joint discussion by women and clinicians. In each site, the intervention was delivered by the midwife and obstetric ‘opinion leaders,’ supported by researchers from the OptiBIRTH team.

A checklist was developed to assess, monitor and evaluate adherence to the intervention in each site, and was applied twice, once during the pilot phase and once during the main trial. Details of the attendance of clinicians at educational sessions, and women at the antenatal classes, the use of online resources and adherence to the intervention were monitored during the trial, in each intervention site [27]. Sites randomised to the control group followed the usual practice for that unit.

### Data collection for recruited women

Women in each site were screened for eligibility for OptiBIRTH at their first visit, using a pre-designed Trial Screening and Register Form. Women judged eligible were informed of the study verbally and received a study pack, which included a detailed information leaflet and consent form. On receipt of the signed consent form, women were contacted by the midwife opinion lead (MOL) for the site and provided with further details on accessing the trial processes (intervention sites only). Although all eligible women could be considered to have been randomised and we used site-level routine data for the primary analyses (see below), individual data could only be used for women who gave their consent. As is expected in an unblinded cluster randomised trial, this can lead to differences between the women who joined the trial in the intervention and control sites, the impact of which is considered through adjusted analyses below.

Women gave their consent to participate in the trial at one of two levels:

1. Full participation: if they were in an intervention site and wanted to attend the OptiBIRTH antenatal classes and, if desired, access the online resources. In both intervention and control sites, a woman choosing full participation agreed to complete health surveys and a diary recording her expenses for healthcare services used, and gave us permission to access the healthcare records for herself and her baby.
2. Routine data only: this gave us permission to access the personal healthcare records for the woman and her baby. In the intervention sites, these women preferred not to attend the antenatal classes or access the online resources and, in both intervention and control sites, they did not wish to complete the health surveys and diary of healthcare expenses.

Ethical approval was granted by the Faculty of Health Sciences, Trinity College Dublin, Ireland and Research Ethics Committees for all participating sites in each country (Additional file 1).

All outcome data on recruited women were collected using pre-designed data forms. These included self-report of antenatal and postnatal health and healthcare resource use and expenditure surveys, and clinician-reported labour and birth outcome data. Data from hospital records of participating women were collected at each site by the MOL and submitted to the trial and data management centre encrypted. To identify and exclude errors, each country’s researchers checked 5 to 10% of the data collected and entered, every quarter. For the hospital-level data, the annual statistics for each site were checked to extract the mode of birth for all eligible women who gave birth at that site in the year before the trial (2012) and in the final year of the trial (2016), This paper reports the analyses for these routinely collected data on labour and birth. Subsequent papers will report analyses of the data collected via the antenatal and postnatal questionnaires, including economic analyses of resource use.

### Outcome measures

#### Primary outcome for site-level data

The pre-specified primary outcome for the trial is the proportion of women with one previous CS who have a vaginal birth in each site comparing the calendar year before the trial (2012) versus the final year of the trial (2016) to assess the sustainability of any intervention effect at site level.

#### Secondary outcomes for recruited women, presented in this paper

Maternal outcomes, collected at or shortly after birth, are:

- Labour onset (spontaneous, induced, etc.)
- Acceleration of labour (artificial rupture of membranes, oxytocin use)
- Mode of birth

Neonatal outcomes, collected during pregnancy and at, or shortly after, birth are:

- Fetal demise during pregnancy (miscarriage or intrauterine death after 24 weeks gestation)
- Gestational age at birth
- Admission to neonatal intensive care unit (NICU)
- Neonatal mortality

Additional secondary outcomes (health economics, quality of life) will be presented in subsequent papers, in accordance with the published protocol [23].

### Sample size assumptions and estimates

We performed a sample size calculation to estimate the number of women who would need to be recruited in each site to detect the desired effect on VBAC (primary outcome) and to provide data on other antenatal and postnatal factors and outcomes. This sample size would be smaller than that available from routine data – i.e., the number of women with one previous CS who would give birth in each site in the year prior to the trial, and the final year of the trial, to be used for the primary outcome analysis. To allow for clustering, an estimate of the sample size for an individually randomised trial was adjusted and inflated by the design effect given by 1 + (ñ-1) ρ, where ñ was the average cluster size and ρ was the estimated intra-class correlation coefficient (ICC) for this study. This sample size calculation used a background proportion of successful VBAC of 25% and an ICC of 0.05. It showed that 12 sites would be required, each containing 120 participating women (840 women in the intervention group and 840 women in the control group), to detect a 15-percentage point difference in successful VBAC (i.e. an absolute increase from 25% in the control group to 40% in the intervention group), with power of at least 80% and an alpha level of 0**.**05. If the true ICC values are less than 0**.**05, the power of the study will increase. To allow for a loss to follow-up of up to 20% of women and the possibility that one site per country would drop out of the trial, 16 maternity units were randomised.

### Data analysis

The primary analysis was to test the effect of OptiBIRTH at site level by comparing VBAC proportions in the intervention and control sites in the calendar year 2016, while adjusting for differences between countries and sites at baseline (i.e., during 2012) and for clustering due to intervention allocation at the site level. The first analysis compared the difference in VBAC proportions aggregated at the site level, and the second analysis compared the difference in VBAC proportions at the individual level. A generalised linear mixed model for a binary response was used for both analyses to model the effect of treatment allocation (i.e. OptiBIRTH intervention versus control), adjusting for baseline VBAC proportion at site level and country of the site, with random intercepts included to account for the variability due to clustering by site. When modelling the data available from those women who participated in OptiBIRTH, we also report results from a generalised linear mixed model with adjustment for baseline VBAC proportion at site level, baseline characteristics of mother’s body mass index (kg/m^2^), age (years), previous vaginal birth before caesarean (yes/no), previous VBAC (yes/no) and gestational age of infant (weeks) with random effects for site and country.

Odds ratios (OR) and corresponding 95% confidence intervals (CI) and *p*-values are reported. All analyses were carried out using R version 3.1 and the lme4 package [28] for generalised linear mixed effects models.

We also analysed data for the women who agreed to take part in the trial and their babies, to assess the effects of the intervention on the secondary outcomes listed above. These were done as fixed-effect meta-analyses of the six randomised comparisons of the sites without adjustment for baseline characteristics, with a sensitivity analysis using the random effects model. They included pre-specified subgroup analyses for (1) whether the woman had had a vaginal birth before her previous CS (yes, no), (2) whether the woman had had a vaginal birth between her previous CS and her current pregnancy (yes, no), (3) age at baseline (< 40 years, ≥40 years), (4) body mass index (BMI) at baseline (< 25 kg/m^2^, 25–29**.**99 kg/m^2^, ≥30 kg/m^2^), and (5) gestational age at baseline (< 30 weeks, 30–32 weeks, ≥33 weeks). Women recruited at ≥33 weeks gestation were categorised as “late” recruits who we assumed might benefit less from the intervention than those who were recruited earlier (< 33 weeks gestation), because of the shorter exposure to the intervention or less opportunity to influence decisions about VBAC. To assess the potential impact of baseline imbalances in this unblinded, cluster randomised trial, sensitivity analyses were done for the overall results for recruited women, in which adjustments were made for baseline characteristics: (1) (2) (4) and (5) above.

### Data monitoring committee

In May 2015, a Data Monitoring Committee (DMC) conducted an interim analysis of data from 700 women who had birthed at that point. The DMC were provided with unblinded data on the VBAC rate at each site in the year before the trial began, the number of women recruited in each site and the proportion of those who had birthed vaginally. Based on the evidence presented at that time, the DMC recommended that the trial should continue.

#### Role of the funding source

The funding body had no input into study design, data collection, analysis, and interpretation, the writing of the report or the decision to submit the paper for publication.

### Recruitment to the main trial

**Fig. 1:**
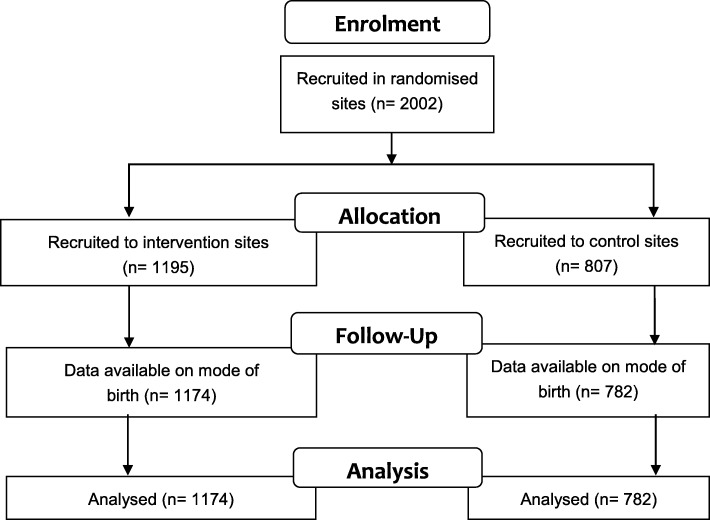
Flow diagram for recruited participants

Women were recruited to the main OptiBIRTH trial from 1 April 2014 in Ireland (*n* = 622) and from 1 May 2014 in Germany (*n* = 755) and Italy (*n* = 625). Most sites closed when they reached their target of 120 participants, during March to May 2015. Formal closure of recruitment was on 31 October 2015. The data set was closed for the purposes of this paper on 9 March 2018. Across the 15 sites, 5674 women (intervention: 2518; control: 3156) who had had one previous CS birthed in 2012, compared to 5284 women (intervention: 2432; control: 2852) in 2016. In the main trial, 2002 women were recruited across the 15 sites in the three countries (Fig. 1).

A total of 1831 women agreed to full participation (intervention: 1073; control: 758), and a further 171 agreed to partial participation (allowing use of their birthing data only) (intervention: 122; control: 49). Baseline data were available on all 2002 women (intervention: 1195; control: 807) who had agreed to take part and some birthing data were available for 1956 (97**.**7%) (intervention: 1174 (98**.**2%); control: 782 (96**.**9%)) of these (Additional files 2, 3, 4).

Table 1 shows that there were statistically significant imbalances between the intervention and control groups for women who had VBAC between their previous CS and their OptiBIRTH pregnancy in Italy, and gestational age at recruitment in Germany. Our adjusted analyses investigate the effect of all imbalances in baseline variables on the findings of the trial, regardless of the statistical significance of the imbalance.

**Table 1:** Baseline characteristics of all women recruited to the trial

Characteristic	Maternity unit or country (number of recruited women)	Intervention sites	Control sites	Odds ratio [95% CI] or *Mean Difference [95% CI]*
**Number recruited**	**Trial as a whole**	1195	807	
	**Germany**	466	289	
	**Ireland**	368	254	
	**Italy**	361	264	
**Any prior vaginal birth (before previous CS)**	**Trial as a whole**	104 (8**.**7%)	54 (6**.**7%)	1**.**30 [0**.**92, 1**.**83]
	**Germany**	28 (6**.**0%)	16 (5**.**5%)	0**.**91 [0**.**48, 1**.**74]
	**Ireland**	58 (15**.**8%)	30 (11**.**8%)	1**.**37 [0**.**86, 2**.**18]
	**Italy**	18 (5**.**0%)	8 (3**.**0%)	1**.**94 [0**.**83, 4**.**56]
**Any prior VBAC**	**Trial as a whole**	75 (6**.**3%)	32 (4**.**0%)	1**.**52 [0**.**99, 2**.**33]
	**Germany**	26 (5**.**6%)	18 (6**.**2%)	0**.**91 [0**.**48, 1**.**71]
	**Ireland**	32 (8**.**7%)	13 (5**.**1%)	1**.**56 [0**.**80, 3**.**06]
	**Italy**	17 (4**.**7%)	1 (0**.**4%)	8**.**94 [1**.**62, 49**.**27]
**Gestational age at recruitment (mean, weeks)**^a^	**Trial as a whole**	26**.**3 (9**.**4)	24**.**8 (10**.**5)	1**.**15 [0**.**42, 1**.**88]
	**Germany**	32**.**7 (5**.**6)	28**.**3 (11**.**4)	4**.**40 [2**.**99, 5**.**81]
	**Ireland**	19**.**6 (7**.**3)	19**.**1 (5**.**3)	0**.**50 [−0**.**49, 1**.**49]
	**Italy**	24**.**8 (9**.**8)	26**.**4 (11**.**1)	−1**.**60 [−3**.**28, 0**.**08]
**BMI at recruitment (mean)**^b^	**Trial as a whole**	25**.**8 (5**.**1)	27**.**0 (5**.**5)	−1**.**26 [− 1**.**73, −0**.**79]
	**Germany**	25**.**6 (5**.**5)	26**.**2 (5**.**3)	−0**.**60 [−1**.**39, 0**.**19]
	**Ireland**	26**.**6 (4**.**9)	27**.**2 (5**.**8)	−0**.**60 [−1**.**47, 0**.**27]
	**Italy**	25**.**2 (4**.**8)	27**.**7 (5**.**2)	−2**.**50 [−3**.**30, −1**.**70]
**Age at recruitment (mean, years)**^c^	**Trial as a whole**	34**.**0 (4**.**5)	34**.**2 (4**.**7)	−0**.**27 [− 0**.**67, 0**.**14]
	**Germany**	34**.**1 (4**.**3)	33**.**8 (4**.**7)	0**.**30 [−0**.**37, 0**.**97]
	**Ireland**	33**.**0 (4**.**2)	33**.**5 (4**.**4)	−0**.**50 [−1**.**19, 0**.**19]
	**Italy**	34**.**8 (4**.**8)	35**.**5 (4**.**6)	−0**.**70 [−1**.**44, 0**.**04]

^a^. Gestational age at baseline is missing for 4 women (intervention: 2; control: 2)

^b^. BMI at baseline is missing for 139 women (intervention: 86; control: 53)

^c^. Age at baseline is missing for 4 women (intervention: 0; control: 4)

## Results

The results comparing the effect of OptiBIRTH are presented initially for site-level comparisons followed by comparisons using individual-level data.

### Analysis at site level

**Fig. 2:**
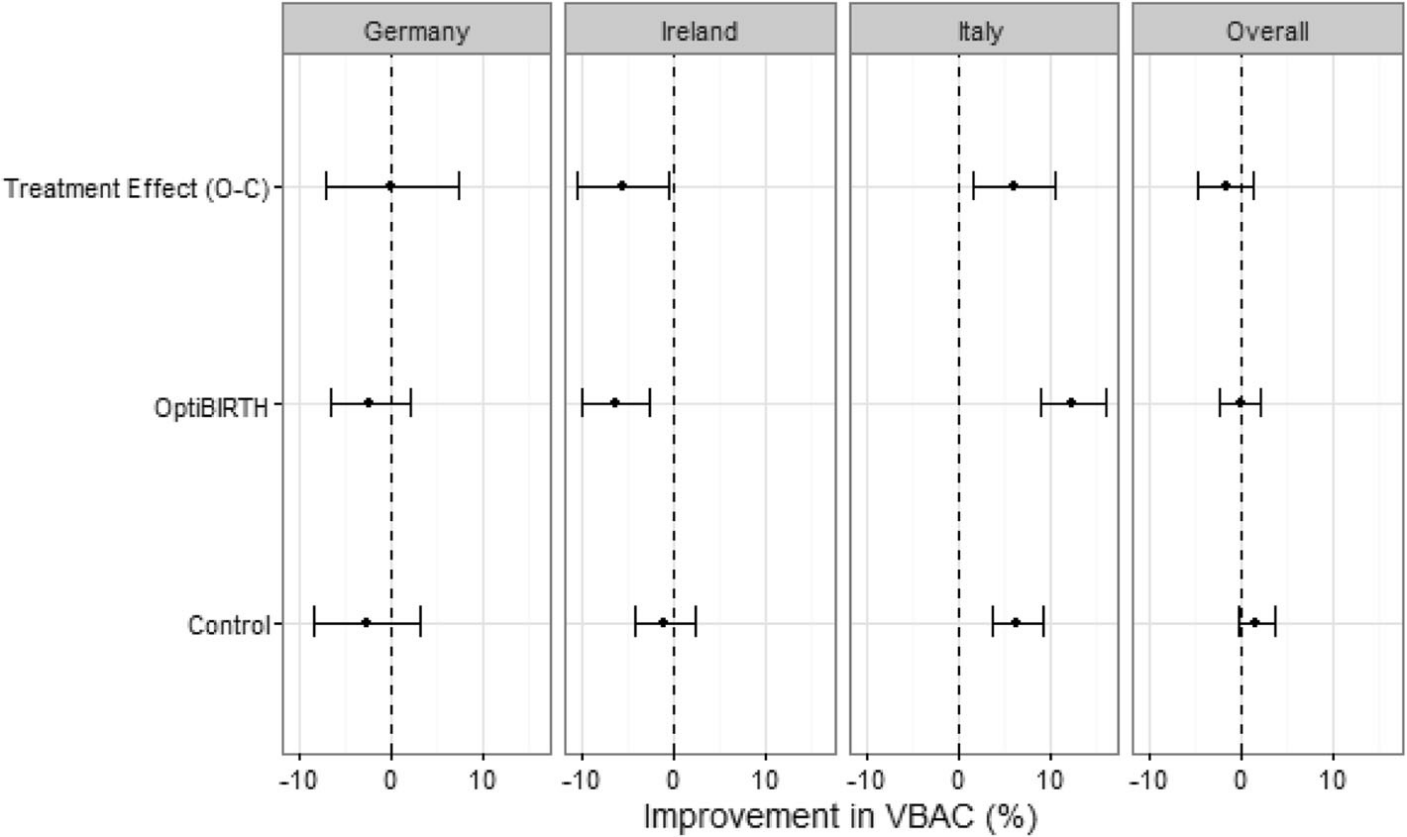
Changes in site-level VBAC rates for 2012 compared to 2015, within countries and overall (adjusted)

The changes in VBAC proportion for each allocation group over time overall, by country, are given in Table 2 and Fig. 2, while a breakdown by site is given in Table 3. There was a 4.02% increase in VBAC proportions among control sites while there was a 0.5% decrease among OptiBIRTH sites between 2012 and 2016.

**Table 2:** VBAC change from 2012 to 2016 by allocation and country

Country	Allocation	Births ‘12 (n)	VBAC ‘12 (n)	VBAC ‘12 (%)	Births ‘16 (n)	VBAC ‘16 (n)	VBAC ‘16 (%)	VBAC (% improvement)
	Control	3156	576	18.25	2852	635	22.27	4.02
	Intervention	2518	645	25.62	2432	611	25.12	−0.50
Germany	Control	473	129	27.27	585	159	27.18	−0.09
	Intervention	733	247	33.70	1030	281	27.28	−6.42
Ireland	Control	1408	312	22.16	1281	266	20.77	−1.39
	Intervention	1049	337	32.13	922	236	25.60	−6.53
Italy	Control	1275	135	10.59	986	210	21.30	10.71
	Intervention	736	61	8.29	480	94	19.58	11.29

**Table 3:** Change in VBAC proportion from 2012 to 2016 by Country and site

Country	Allocation	Site	Births 2012 (n)	VBAC 2012 (n)	VBAC 2012 (%)	Births 2016 (n)	VBAC 2016 (n)	VBAC 2016 (%)	VBAC (% improvement)
Germany	Control	1	284	73	25.70	365	81	22.19	−3.51
		2	189	56	29.63	220	78	35.45	5.82
	Intervention	3	288	99	34.38	420	107	25.48	−8.90
		4	445	148	33.26	610	174	28.52	−4.73
Ireland	Control	5	889	192	21.60	861	186	21.60	0.01
		6	519	120	23.12	420	80	19.05	−4.07
	Intervention	7	279	104	37.28	214	63	29.44	−7.84
		8	547	153	27.97	549	122	22.22	−5.75
		9	223	80	35.87	159	51	32.08	−3.80
Italy	Control	10	224	4	1.79	233	24	10.30	8.51
		11	1051	131	12.46	753	186	24.70	12.24
	Intervention	12	77	26	33.77	112	32	28.57	−5.19
		13	212	11	5.19	82	22	26.83	21.64
		14	447	24	5.37	286	40	13.99	8.62

There were decreases in VBAC proportions in both control (1.39%) and intervention (6.53%) sites in Ireland, and increases in both control (10.71%) and intervention (11.29%) sites in Italy (i.e. intervention sites had better improvements than control in Italy). The largest improvement and reduction in VBAC proportions occurred in intervention sites, with a 21.64% increase in Italy (Site 13) and an 8.9% decrease in Germany (Site 3) (Table 3).

Overall, the adjusted analyses (Table 4) showed no evidence of a significant intervention effect while adjusting for differences between the intervention and control groups at baseline (year 2012) due to randomisation at site level and for homogeneity in the VBAC rates at sites in countries (odds ratio: 0**.**87, 95% CI: 0**.**67, 1**.**14, *p* = 0**.**32).

**Table 4:** Generalised mixed effect logistic model adjusted for 2012 baseline VBAC rate and country, with random intercept for site

	Odds Ratio	95% CI	*p*-value
OptiBIRTH v Control	0.87	(0.67, 1.14)	0.32
VBAC proportion in 2012 (%)	1.04	(1.02, 1.06)	< 0.001
Germany v Ireland	1.09	(0.85, 1.39)	0.49
Italy v Ireland	1.44	(0.98, 2.12)	0.06

ICC for Site = 0.0057

There was evidence that the proportion of VBAC in 2012 was a useful predictor of VBAC proportions in 2016 (odds ratio: 1.04, 95% CI: 1.02, 1.06, *p* <  0.001), a possible higher odds of VBAC in Italy compared to Ireland (odds ratio: 1.44, 95% CI: 0.98, 2.12, *p* = 0.06), no evidence of a significant difference between Ireland and Germany (odds ratio = 1.09, 95% CI: 0.85, 1.39, *p* = 0.49) and between Italy and Germany (odds ratio = 0.32, 95% CI: 0.85, 2.04, *p* = 0.21). The intracluster correlation due to clustering at the site level was estimated to be 0.006.

### Analysis at individual level

There was no evidence of a significant OptiBIRTH effect (OR 1.09, 95% CI 0.76 to 1.56; *p* = 0.66) after adjustment for covariates (Table 5) based on the analysis of the individual level data from the 1956 full participants. The odds of having a VBAC decreased with increasing BMI (OR 0.94, 95% CI 0.92 to 0.96; *p* <  0.001) and increased with increasing gestational age at birth (OR 1.02, 95% CI 1.01 to 1.03; *p* = 0.02). Neither a mother’s age nor proportion of VBAC in 2012 were useful predictors of VBAC proportions in 2016 when adjusting for baseline covariates. Having had a previous VBAC significantly increased the odds of a subsequent VBAC (OR 0.42, 95% CI 0.30 to 0.60; *p* < 0.001), while having had a vaginal birth before a CS significantly decreased the odds of a subsequent VBAC (OR 7.18, 95% CI 4.41 to 11.69; *p* < 0.001). Italy had a lower VBAC proportion than Ireland (OR 0.6, 95% CI 0.36 to 1.01; *p* = 0.05), while Germany had a higher proportion (OR 1.56, 95% CI 1.11 to 2.2; *p* = 0.01) than Ireland.

**Table 5:** Logistic model of VBAC in 2016 for *n* = 1956 with covariate data available, adjusted for 2012 VBAC rate and covariates with random intercept for site

	Odds Ratio	95% CI	*p*-value
OptiBIRTH	1.09	0.76, 1.56	0.66
VBAC 2012 (%)	1.00	0.97, 1.02	0.76
BMI (kg/m2)	0.94	0.92, 0.96	< 0.001
Age (years)	0.99	0.96, 1.01	0.24
Gestational Age (weeks)	1.02	1.01, 1.03	0.02
VB before CS	0.42	0.30, 0.60	< 0.001
Previous VBAC	7.18	4.41, 11.69	< 0.001
Germany v Ireland	1.43	0.96, 2.12	0.08
Italy v Ireland	0.58	0.34, 1	0.05

#### Birth outcomes, using data for recruited women

##### Mode of birth

**Fig. 3:**
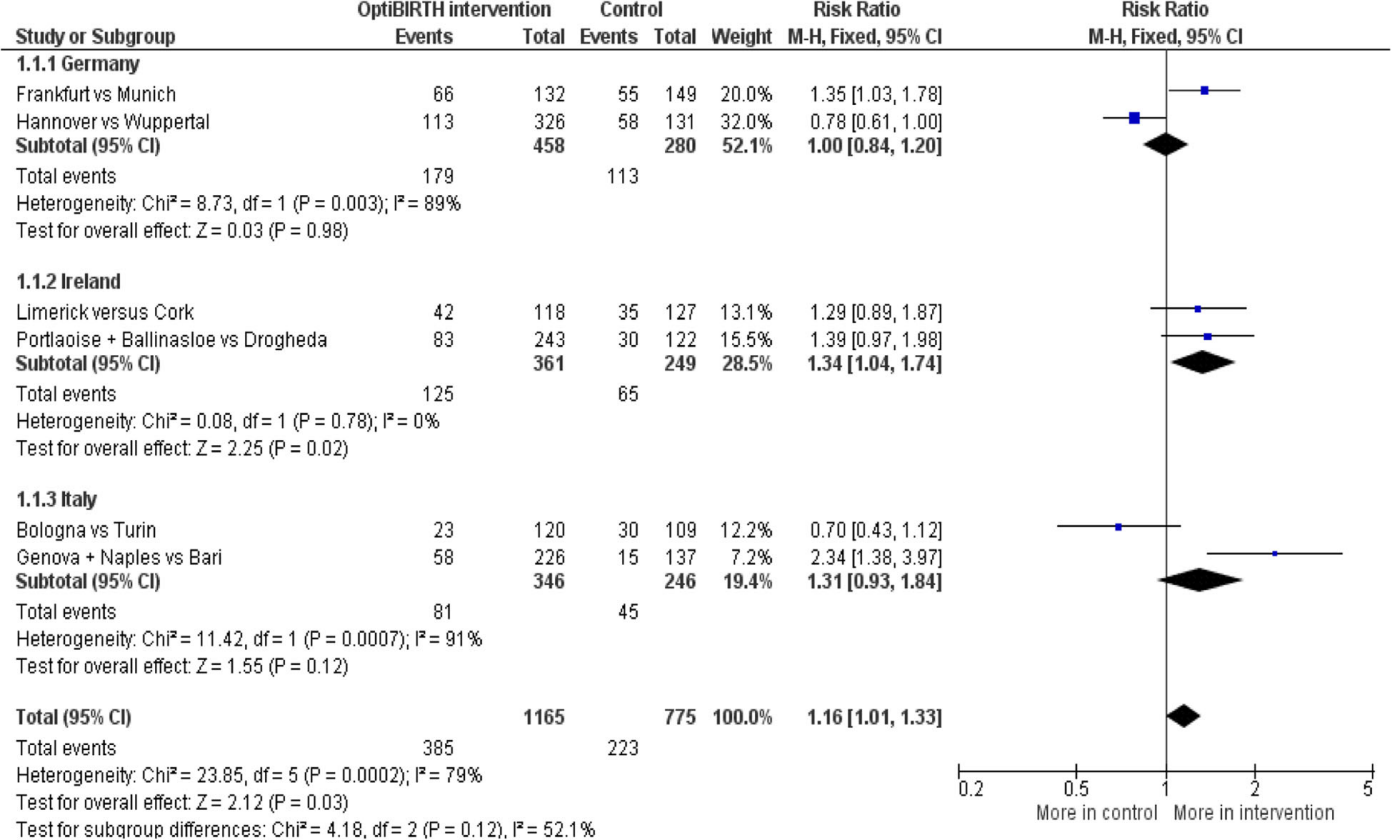
VBAC for the recruited women with data on mode of birth, by randomised comparison, within countries and overall

Table 6 shows the mode of birth for women who agreed to join the trial in each of the countries and for the trial overall. No birth data are available for 46 of the 2002 recruited women (for example, because they moved to a different area or gave birth elsewhere), and mode of birth is not available for a further 16 women who had a miscarriage/intrauterine death at less than 24 weeks gestation (*n* = 8) or more than 24 weeks (3) or for whom the mode of birth was missing at the time of data lockdown (5). Therefore, of the 1940 recruited women with known mode of birth, 385 (33**.**0% of 1165) in the intervention group and 223 (28**.**8% of 775) in the control group had a VBAC, but there is substantial statistical heterogeneity across the six randomised comparisons (Fig. 3).

**Table 6:** Mode of birth for the 1940 recruited women with known mode of birth

Mode of birth	Country	Intervention sites	Control sites	Risk ratio [95% CI]
**All births**	**Trial as a whole**	1165	775	
	**Germany**	458	280	
	**Ireland**	361	249	
	**Italy**	346	246	
**VBAC (% of births)**	**Trial as a whole**	385 (33**.**0)	223 (28**.**8)	1**.**16 [1**.**01, 1**.**33]
	**Germany**	179 (39**.**1)	113 (40**.**4)	1**.**00 [0**.**84, 1**.**20]
	**Ireland**	125 (34**.**6)	65 (26**.**1)	1**.**34 [1**.**04, 1**.**74]
	**Italy**	81 (23**.**4)	45 (18**.**3)	1**.**31 [0**.**93, 1**.**84]
**Spontaneous vaginal (% of births)**	**Trial as a whole**	306 (26**.**3)	170 (21**.**9)	1**.**22 [1**.**04, 1**.**44]
	**Germany**	150 (32**.**8)	92 (32**.**9)	1**.**06 [0**.**86, 1**.**31]
	**Ireland**	84 (23**.**3)	38 (15**.**3)	1**.**52 [1**.**07, 2**.**15]
	**Italy**	72 (20**.**8)	40 (16**.**3)	1**.**31 [0**.**91, 1**.**90]
**Ventouse (% of births)**	**Trial as a whole**	78 (6**.**7)	47 (6**.**1)	1**.**08 [0**.**76, 1**.**53]
	**Germany**	29 (6**.**3)	21 (7**.**5)	0**.**77 [0**.**45, 1**.**32]
	**Ireland**	40 (11**.**1)	21 (8**.**4)	1**.**38 [0**.**82, 2**.**34]
	**Italy**	9 (2**.**6)	5 (2**.**0)	1**.**28 [0**.**42, 3**.**90]
**Forceps (% of births)**	**Trial as a whole**	1 (0**.**1)	6 (0**.**8)	0**.**16 [0**.**03, 0**.**99]
	**Germany**	0 (0)	0 (0)	No data
	**Ireland**	1 (0**.**3)	6 (2**.**4)	0**.**16 [0**.**03, 0**.**99]
	**Italy**	0 (0)	0 (0)	No data
**Elective CS (% of births)**	**Trial as a whole**	504 (43**.**3)	393 (50**.**7)	0**.**85 [0**.**77, 0**.**93]
	**Germany**	178 (38**.**9)	98 (35**.**0)	1**.**05 [0**.**85, 1**.**30]
	**Ireland**	119 (33**.**0)	131 (52**.**6)	0**.**65 [0**.**54, 0**.**78]
	**Italy**	207 (59**.**8)	164 (66**.**7)	0**.**89 [0**.**78, 1**.**00]
**Emergency CS (not in labour) (% of births)**	**Trial as a whole**	141 (12**.**1)	57 (7**.**4)	1**.**64 [1**.**22, 2**.**21]
	**Germany**	41 (9**.**0)	13 (4**.**6)	1**.**88 [1**.**02, 3**.**46]
	**Ireland**	61 (16**.**9)	17 (6**.**8)	2**.**27 [1**.**35, 3**.**83]
	**Italy**	39 (11**.**3)	27 (11**.**0)	1**.**10 [0**.**70, 1**.**74]
**Emergency CS (in labour) (% of births)**	**Trial as a whole**	135 (11**.**6)	102 (13**.**2)	0**.**88 [0**.**68, 1**.**13]
	**Germany**	60 (13**.**1)	56 (20**.**0)	0**.**69 [0**.**48, 0**.**98]
	**Ireland**	56 (15**.**5)	36 (14**.**5)	1**.**07 [0**.**70, 1**.**64]
	**Italy**	19 (5**.**5)	10 (4**.**1)	1**.**27 [0**.**61, 2**.**66]

There is a statistically significant relative increase in VBAC of 16% for the intervention group compared to the control group (risk ratio (RR): 1**.**16; 95% CI: 1**.**01 to 1**.**33; *p* = 0**.**03), with an absolute increase of 5% (95% CI: 0 to 9%) for the intervention group compared to the control group. However, when this analysis is adjusted for the baseline characteristics, the difference becomes non-significant. It is also not significant if the random effects model is used for the meta-analysis (RR: 1**.**19; 95% CI: 0**.**88 to 1**.**62; *p* = 0**.**28).

##### Onset of labour

Of the 1956 women for whom some birth data are available, onset of labour was spontaneous in 445 (37**.**9% of 1174) in the intervention group and 281 (35**.**9% of 782) in the control (RR: 1**.**07; 95% CI: 0**.**95 to 1**.**21; *p* = 0**.**25), a non-significant difference. Labour was induced for 82 women (10**.**5%) in the intervention group and 49 (6**.**2%) in the control. The remaining women had CS without going into labour, 647 (55**.**1%) in the intervention group and 452 (57**.**8%) in the control group.

Of the 857 women who laboured (for all of whom we have birth data), labour onset was spontaneous in 445 (84**.**4% of 527) in the intervention group and 281 (85**.**2% of 330) in the control (Table 7) (RR: 0**.**99; 95% CI: 0**.**93 to 1**.**05; *p* = 0**.**62), a non-significant difference. Labour was induced for the other 82 (15**.**6%) in the intervention group and 49 (14**.**8%) in the control group who laboured.

**Table 7:** Spontaneous onset of labour for the recruited women who went into labour

Mode of birth	Country	Intervention sites	Control sites	Risk ratio [95% CI]
**Spontaneous onset of labour (% of women who went into labour)**	**Trial as a whole**	445 (84**.**4)	281 (85**.**2)	0**.**99 [0**.**93, 1**.**05]
	**Germany**	185 (77**.**1)	136 (79**.**5)	0**.**98 [0**.**88, 1**.**08]
	**Ireland**	157 (86**.**3)	92 (89**.**3)	0**.**96 [0**.**87, 1**.**06]
	**Italy**	103 (98**.**1)	53 (94**.**6)	1**.**04 [0**.**97, 1**.**11]

Of the 857 women who laboured, labour was accelerated in 226 women (42**.**9% of 527) in the intervention group and 146 (44**.**2% of 330) in the control group (RR: 0**.**99; 95% CI: 0**.**85 to 1**.**16; *p* = 0**.**88), a non-significant difference.

##### Uterine rupture

Data on all reported uterine ruptures were reviewed, to distinguish between scar dehiscence and full uterine rupture; there were two full uterine ruptures in the main trial (one in each group), one following an induced labour, the other spontaneous. Both women and babies were discharged home well by day 5 and day 4, respectively.

##### Subgroup analyses for maternal outcomes

Caution is needed in the interpretation of subgroup analyses, when the overall result is not statistically significant [29, 30]. However, for completeness, Table 8 presents the main maternal outcomes within the pre-specified subgroups. This shows variable effects in different subgroups. However, given the number of such analyses, the relatively small number of women in some subgroups, and the challenges of recruiting participants in a cluster randomised trial when the allocation cannot be concealed, there is no strong evidence of clear benefit or harm for the OptiBIRTH intervention in any of the subgroups. Given the possibility that women who were recruited late to the trial (at or beyond week 33 of their pregnancy) might lead to an underestimate of any effect of the intervention, a sensitivity analysis was done excluding such women. This found a RR of 1**.**08 (95% CI: 0**.**88 to 1**.**31, *p* = 0**.**46) for VBAC comparing the intervention and control groups.

**Table 8:** Labour and birth outcomes for the recruited women, by subgroups

Outcome	Subgroup (relevant births in intervention; control groups)	Intervention	Control	Risk ratio [95% CI]
**VBAC (% of all births with known mode of birth that were VBAC)**^a^	**Trial as a whole (1165; 775)**	385 (33**.**0)	223 (28.8)	1**.**16 [1**.**01, 1**.**33]
	Germany (458; 280)	179 (39**.**1)	113 (40**.**4)	1**.**00 [0**.**84, 1**.**20]
	Ireland (361; 241)	125 (34**.**6)	65 (26**.**1)	1**.**34 [1**.**04, 1**.**74]
	Italy (346; 246)	81 (23**.**4)	45 (18**.**3)	1**.**31 [0**.**93, 1**.**84]
	Vaginal birth before previous CS (104; 54)	51 (49**.**0)	26 (48**.**1)	0**.**99 [0**.**70, 1**.**40]
	No vaginal births before previous CS (1061; 721)	334 (31**.**5)	197 (27**.**3)	1**.**17 [1**.**01, 1**.**36]
	Prior VBAC (74; 32)	54 (73**.**0)	25 (78**.**1)	0**.**98 [0**.**77, 1**.**25]
	No prior VBAC (1091; 743)	331 (30**.**3)	198 (26**.**6)	1**.**15 [0**.**99, 1**.**34]
	Recruitment at < 30 weeks (621; 459)	178 (28**.**7)	123 (26**.**8)	1**.**14 [0**.**92, 1**.**41]
	Recruitment at 30–32 weeks (104; 44)	28 (26**.**9)	15 (34**.**1)	0**.**65 [0**.**38, 1**.**11]
	Recruitment at ≥33 weeks (438; 270)	178 (40**.**6)	85 (31**.**5)	1**.**21 [0**.**99, 1**.**48]
	BMI (< 25.00) (555; 307)	208 (37**.**5)	113 (36**.**8)	1**.**06 [0**.**89, 1**.**26]
	BMI (25–29.99) (347; 268)	103 (29**.**7)	72 (26**.**9)	1**.**06 [0**.**81, 1**.**38]
	BMI (> 29.99) (201; 175)	52 (25**.**9)	33 (18**.**9)	1**.**34 [0**.**91, 1**.**96]
	Age (< 40 years) (1074; 701)	354 (33**.**0)	210 (30**.**0)	1**.**11 [0**.**97, 1**.**28]
	Age (≥40 years) (91; 71)	31 (34**.**1)	13 (18**.**3)	1**.**96 [1**.**10, 3**.**49]
**Spontaneous onset of labour (% of all women who went into labour who had a spontaneous onset of labour)**^b^	**Trial as a whole** (527; 330)	445 (84**.**4)	281 (85**.**2)	0**.**99 [0**.**93, 1**.**05]
	Germany (240; 171)	185 (77**.**1)	136 (79**.**5)	0**.**98 [0**.**88, 1**.**08]
	Ireland (182; 103)	157 (86**.**3)	92 (89**.**3)	0**.**96 [0**.**87, 1**.**06]
	Italy (105; 56)	103 (98**.**1)	53 (94**.**6)	1**.**04 [0**.**97, 1**.**11]
	Vaginal birth before previous CS (57; 50)	50 (87**.**7)	24 (80**.**0)	1**.**07 [0**.**83, 1**.**38]
	No vaginal births before previous CS (470; 300)	395 (84**.**0)	257 (85**.**7)	0**.**98 [0**.**92, 1**.**04]
	Prior VBAC (56; 29)	44 (78**.**6)	21 (72**.**4)	1**.**02 [0**.**78, 1**.**32]
	No prior VBAC (471; 301)	401 (85**.**1)	260 (86**.**4)	0**.**98 [0**.**92, 1**.**04]
	Recruitment at < 30 weeks (255; 186)	226 (88**.**6)	161 (86**.**6)	1**.**01 [0**.**92, 1**.**09]
	Recruitment at 30–32 weeks (42; 22)	33 (78**.**6)	19 (86**.**4)	0**.**97 [0**.**73, 1**.**30]
	Recruitment at ≥33 weeks (229; 122)	186 (81**.**2)	101 (82**.**8)	0**.**98 [0**.**88, 1**.**08]
	BMI (< 25.00) (277; 158)	237 (85**.**6)	137 (86**.**7)	0**.**99 [0**.**91, 1**.**08]
	BMI (25–29.99) (151; 106)	127 (84**.**1)	90 (84**.**9)	0**.**97 [0**.**86, 1**.**09]
	BMI (> 29.99) (76; 57)	60 (78**.**9)	46 (80**.**7)	0**.**95 [0**.**78, 1**.**14]
	Age (< 40 years) (489; 311)	414 (84**.**7)	265 (85**.**2)	0**.**99 [0**.**93, 1**.**05]
	Age (≥40 years) (38; 19)	31 (81**.**6)	16 (84**.**2)	1**.**04 [0**.**82, 1**.**31]

^a^. The following data are missing for women who gave birth: gestational age at baseline is missing for 1 women (intervention), BMI at baseline is missing for 27 women (intervention: 22; control: 5)

^b^. The following data are missing for women who went into labour: BMI at baseline is missing for 29 women (intervention: 21; control: 8)

#### Antenatal and neonatal outcomes, using data for recruited women

Table 9 shows neonatal outcomes for women who agreed to join the trial. These data use the birth data that are available for 1956 (97**.**7%) of the 2002 recruited women.

**Table 9:** Antenatal and neonatal outcomes

Outcome	Country (recruited women relevant to each subgroup with birth data available in intervention; control groups)	Intervention sites	Control sites	Risk ratio [95% CI] or *Mean Difference [95% CI]*
**Intrauterine death before 24 weeks (% with birthing data)**	Trial as a whole (1174; 782)	7 (0**.**6)	3 (0**.**4)	1**.**22 [0**.**36, 4**.**11]
	Germany (458; 282)	0 (0**.**0)	1 (0**.**4)	0**.**38 [0**.**02, 9**.**27]
	Ireland (365; 250)	6 (1**.**6)	0 (0)	6**.**45 [0**.**37, 113**.**52]
	Italy (351; 250)	1 (0**.**3)	2 (0**.**8)	0**.**50 [0**.**07, 3**.**36]
**Intrauterine death after 24 weeks (% with birthing data)**	Trial as a whole (1174; 782)	4 (0**.**2)	2 (0**.**2)	1**.**16 [0**.**28, 4**.**83]
	Germany (458; 282)	1 (0**.**2)	1 (0**.**4)	0**.**66 [0**.**08, 5**.**78]
	Ireland (365; 250)	1 (0**.**3)	1 (0**.**4)	1**.**08 [0**.**07, 17**.**15]
	Italy (351; 250)	2 (0**.**6)	0 (0)	2**.**97 [0**.**14, 61**.**50]
**Neonatal death (% of live-born)**	Trial as a whole (1163; 777)	0 (0)	2 (0**.**3)	0**.**26 [0**.**03, 2**.**34]
	Germany (457; 280)	0 (0)	1 (0**.**4)	0**.**38 [0**.**02, 9**.**15]
	Ireland (358; 249)	0 (0)	1 (0**.**4)	0**.**17 [0**.**01, 4**.**13]
	Italy (348; 248)	0 (0)	0 (0)	No data
**Live-born before 37 weeks (% of live-born)**^a^	Trial as a whole (1163; 777)	62 (5**.**3)	50 (6**.**4)	0**.**81 [0**.**56, 1**.**17]
	Germany (457; 280)	18 (3**.**9)	16 (5**.**7)	0**.**61 [0**.**31, 1**.**21]
	Ireland (358; 249)	19 (5**.**3)	10 (4**.**0)	1**.**23 [0**.**59, 2**.**56]
	Italy (348; 248)	25 (7**.**2)	24 (9.7)	0**.**77 [0**.**45, 1**.**32]
**Gestational age at live birth (mean, SD)**^a^	Trial as a whole (1163; 777)	38**.**9 (1**.**5)	38**.**7 (1**.**8)	*0****.****15 [0****.****00, 0****.****30]*
	Germany (457; 280)	38**.**9 (1**.**4)	39**.**0 (1**.**8)	*−0****.****10 [−0****.****35, 0****.****15]*
	Ireland (358; 249)	39**.**1 (1**.**5)	39**.**0 (1**.**7)	*0****.****10 [−0****.****16, 0****.****36]*
	Italy (348; 248)	38**.**6 (1**.**7)	38**.**1 (1**.**6)	*0****.****50 [0****.****23, 0****.****77]*
**Admission to NICU (% of live-born)**	Trial as a whole (1163; 777)	90 (7**.**7)	63 (8**.**1)	0**.**87 [0**.**64, 1**.**17]
	Germany (457; 280)	34 (7**.**4)	28 (10**.**0)	0**.**58 [0**.**35, 0**.**95]
	Ireland (358; 249)	40 (11**.**2)	28 (11**.**2)	0**.**97 [0**.**62, 1**.**51]
	Italy (348; 248)	16 (4**.**6)	7 (2**.**8)	1**.**70 [0**.**73, 3**.**93]

1. Gestational age at birth is missing for 56 live-born infants (intervention: 25; control: 31)

A total of 16 pregnancies (intervention: 11; control: 5) ended in intrauterine death, 10 before 24 weeks’ gestation (intervention: 7; control: 3) and six after (intervention: 4; control: 2). Taken separately, or combined, there was no significant difference in these deaths between the intervention and control groups. Two of the recruited women experienced a neonatal death, both in the control group, giving total perinatal death figures, after 24 weeks gestation, of four out of 1174 (0**.**34%) for whom some birth data are available in the intervention group and four out of 782 in the control (0**.**51%).

Of the 1940 babies born alive (and for whom we have birth data, excluding the second twin born to a woman in the control group), 62 babies (5**.**3% of 1163) in the intervention group were born before 37 weeks gestation, compared to 50 (6**.**4% of 777) in the control group (Table 6) (RR: 0**.**81; 95% CI: 0**.**56 to 1**.**17; *p* = 0**.**26), a non-statistically significant difference (95% CI: − 3 to 1%). Similarly, there was little difference in mean gestational age at birth between babies in the intervention (mean: 38**.**9 weeks; SD: 1**.**5) and control (mean: 38**.**7 weeks; SD: 1**.**8) groups. This difference of approximately 1 day is of borderline statistical significance in the unadjusted analysis (95% CI: 0**.**07 weeks to 0**.**28 weeks; *p* = 0**.**05).

Of the 1163 live-born babies in the intervention group for whom data are available, 90 (7**.**7%) were admitted to NICU compared to 63 (8**.**1% of 777) in the control (Table 6) (RR: 0**.**87; 95% CI: 0**.**64 to 1**.**17; *p* = 0**.**36), a non-significant difference.

#### Subgroup analyses for neonatal outcomes

For most neonatal outcomes, the small number of events does not warrant dividing the analyses into subgroups using baseline characteristics of the mothers. However, Table 10 shows these subgroup analyses for three outcomes: birth before 37 weeks, gestational age at birth and admission to NICU. As with the subgroup analyses for maternal labour and birth outcomes (Table 8), this shows variable effects in different subgroups, but without any strong evidence of clear benefit or harm for the OptiBIRTH intervention for the babies based on any of the subgroups.

**Table 10:** Neonatal outcomes, by subgroups

Outcome	Subgroup (recruited women relevant to each subgroup with baby data available in intervention; control groups)	Intervention	Control	Risk ratio [95% CI] or *Mean Difference [95% CI]*
**Live-born before 37 weeks (% of live-born)**^a^	**Trial as a whole (1163; 777)**	62 (5**.**3)	50 (6**.**4)	0**.**81 [0**.**56, 1**.**17]
	Germany (457; 280)	18 (3**.**9)	16 (5**.**7)	0**.**61 [0**.**31, 1**.**21]
	Ireland (358; 249)	19 (5**.**3)	10 (4**.**0)	1**.**23 [0**.**59, 2**.**56]
	Italy (348; 248)	25 (7**.**2)	24 (9**.**7)	0**.**77 [0**.**45, 1**.**32]
	Vaginal birth before previous CS (104; 54)	8 (7**.**7)	7 (13**.**0)	0**.**67 [0**.**26, 1**.**71]
	No vaginal births before previous CS (1059; 723)	54 (5**.**1)	43 (5**.**9)	0**.**83 [0**.**56, 1**.**24]
	Prior VBAC (74; 32)	3 (4**.**1)	1 (3**.**1)	0**.**87 [0**.**13, 6**.**02]
	No prior VBAC (1089; 745)	59 (5**.**4)	49 (6**.**6)	0**.**81 [0**.**56, 1**.**17]
	Recruitment at < 30 weeks (619; 461)	44 (7**.**1)	31 (6**.**7)	0**.**98 [0**.**62, 1**.**56]
	Recruitment at 30–32 weeks (104; 44)	7 (6**.**7)	5 (11**.**4)	0**.**73 [0**.**26, 2**.**11]
	Recruitment at ≥33 weeks (438; 270)	11 (2**.**5)	13 (4**.**8)	0**.**51 [0**.**24, 1**.**10]
	BMI (< 25.00) (553; 309)	25 (4**.**5)	21 (6**.**8)	0**.**64 [0**.**37, 1**.**11]
	BMI (25–29.99) (347; 268)	23 (6**.**6)	15 (5**.**6)	1**.**19 [0**.**61, 2**.**33]
	BMI (> 29.99) (202; 175)	10 (5**.**0)	14 (8**.**0)	0**.**77 [0**.**34, 1**.**77]
	Age (< 40 years) (1072; 702)	55 (5**.**1)	40 (5**.**7)	0**.**88 [0**.**59, 1**.**32]
	Age (≥40 years) (91; 72)	7 (7**.**7)	9 (12**.**5)	0**.**63 [0**.**26, 1**.**52]
**Gestational age at birth (mean, SD)**^b^	**Trial as a whole (1163; 777)**	38**.**9 (1**.**5)	38**.**7 (1**.**8)	*0****.****15 [0****.****00, 0****.****30]*
	Germany (457; 280)	38**.**9 (1**.**4)	39**.**0 (1**.**8)	*−0****.****10 [−0****.****35, 0****.****15]*
	Ireland (358; 249)	39**.**1 (1**.**5)	39**.**0 (1**.**7)	*0****.****10 [−0****.****16, 0****.****36]*
	Italy (348; 248)	38**.**6 (1**.**7)	38**.**1 (1**.**6)	*0****.****50 [0****.****23, 0****.****77]*
	Vaginal birth before previous CS (104; 54)	38**.**7 (1**.**9)	38**.**4 (2**.**0)	*0****.****30 [−0****.****35, 0****.****95]*
	No vaginal births before previous CS (1059; 723)	38**.**9 (1**.**5)	38**.**7 (1**.**7)	*0****.****20 [0****.****05, 0****.****35]*
	Prior VBAC (74; 32)	39**.**0 (1**.**5)	39**.**4 (1**.**4)	*−0****.****40 [−0****.****99, 0****.****19]*
	No prior VBAC (1089; 745)	38**.**9 (1**.**6)	38**.**7 (1**.**8)	*0****.****20 [0****.****04, 0****.****36]*
	Recruitment at < 30 weeks (619; 461)	38**.**7 (1**.**7)	38**.**7 (1**.**8)	*0****.****00 [−0****.****21, 0****.****21]*
	Recruitment at 30–32 weeks (104; 44)	38**.**7 (1**.**5)	38**.**3 (1**.**9)	*0****.****40 [−0****.****23, 1****.****03]*
	Recruitment at ≥33 weeks (438; 270)	39**.**1 (1**.**3)	38**.**9 (1**.**5)	*0****.****20 [−0****.****02, 0****.****42]*
	BMI (< 25**.**00) (553; 309)	38**.**9 (1**.**5)	38**.**8 (1**.**7)	*0****.****10 [−0****.****13, 0****.****33]*
	BMI (25–29**.**99) (347; 268)	38**.**9 (1**.**6)	38**.**7 (1**.**7)	*0****.****20 [−0****.****06, 0****.****46]*
	BMI (> 29**.**99) (202; 175)	38**.**8 (1**.**5)	38**.**5 (1**.**9)	*0****.****30 [−0****.****05, 0****.****65]*
	Age (< 40 years) (1072; 702)	38**.**9 (1**.**6)	38**.**8 (1**.**7)	*0****.****10 [−0****.****06, 0****.****26]*
	Age (≥40 years) (91; 72)	38**.**6 (1**.**5)	38**.**3 (1**.**8)	*0****.****30 [−0****.****22, 0****.****82]*
**Admission to NICU (% of live-born)**^c^	**Trial as a whole (1163; 777)**	90 (7**.**7)	63 (8**.**1)	0**.**87 [0**.**64, 1**.**17]
	Germany (457; 280)	34 (7**.**4)	28 (10**.**0)	0**.**58 [0**.**35, 0**.**95]
	Ireland (358; 249)	40 (11**.**2)	28 (11**.**2)	0**.**97 [0**.**62, 1**.**51]
	Italy (348; 248)	16 (4**.**6)	7 (2**.**8)	1**.**70 [0**.**73, 3**.**93]
	Vaginal birth before previous CS (104; 54)	11 (10**.**6)	6 (11**.**1)	0**.**97 [0**.**43, 2**.**21]
	No vaginal births before previous CS (1059; 723)	79 (7**.**5)	57 (7**.**9)	0**.**85 [0**.**62, 1**.**18]
	Prior VBAC (74; 32)	8 (10**.**8)	4 (12**.**5)	0**.**93 [0**.**34, 2**.**58]
	No prior VBAC (1089; 745)	82 (7**.**5)	59 (7**.**9)	0**.**87 [0**.**64, 1**.**20]
	Recruitment at < 30 weeks (619; 462)	60 (9**.**7)	40 (8**.**7)	1**.**08 [0**.**74, 1**.**58]
	Recruitment at 30–32 weeks (104; 44)	6 (5**.**8)	4 (9**.**1)	0**.**68 [0**.**22, 2**.**11]
	Recruitment at ≥33 weeks (438; 270)	23 (5**.**3)	19 (7**.**0)	0**.**63 [0**.**36, 1**.**11]
	BMI (< 25.00) (553; 309)	40 (7**.**2)	25 (8**.**1)	0**.**91 [0**.**56, 1**.**48]
	BMI (25–29.99) (347; 268)	29 (8**.**4)	16 (6**.**0)	0**.**96 [0**.**54, 1**.**70]
	BMI (> 29.99) (202; 175)	17 (8**.**4)	21 (12**.**0)	0**.**56 [0**.**31, 1**.**01]
	Age (< 40 years) (1072; 702)	86 (8**.**0)	60 (8**.**5)	0**.**87 [0**.**64, 1**.**19]
	Age (≥40 years) (91; 72)	4 (4**.**4)	3 (4**.**2)	0**.**72 [0**.**17, 2**.**99]

^a^. Among infants who were live-born before 37 weeks gestation: gestational age at baseline is missing for 1 mother (control), BMI at baseline is missing for 4 mothers (intervention: 4; control: 0), age at baseline is missing for 1 mother (control)

^b^. Among infants who were live-born with gestational age at birth available: gestational age at baseline is missing for 4 mothers (intervention: 2; control: 2), BMI at baseline is missing for 57 mothers (intervention: 57; control: 20)

^c^. Among infants who were admitted to NICU: gestational age at baseline is missing for 1 mother (intervention), BMI at baseline is missing for 5 mothers (intervention: 4; control: 1)

## Discussion

We have demonstrated through this cluster randomised trial that, in these hospital settings in three countries, the OptiBIRTH intervention is feasible and safe. Overall, in this analysis we found no statistically significant difference in the change in the proportion of women having a VBAC between the intervention sites (25**.**6% in 2012 to 25.1% in 2016) and the control sites (18**.**3% in 2012 to 22.3% in 2016). Change in clinical practice is notoriously slow [31], and both clinicians and women need time to become accustomed to new approaches to care. In particular, complex interventions such as that developed for OptiBIRTH require the passage of time and a willingness to engage with them to ensure uptake. A continuance of slow change over the next few years, resulting in an improvement in VBAC rates of even 5% would be clinically significant.

In Italy, where VBAC rates in intervention and control sites were lower pre-trial (8**.**3 and 10**.**6%) compared with Ireland and Germany (32**.**1 and 22**.**2%, and 33**.**7 and 27**.**3%), VBAC rates increased to a greater extent. The overall VBAC rate rose in intervention sites in Italy from 8**.**3% in 2012, to 19**.**6% in 2016, the year after the trial. Thus, in countries or sites where VBAC rates are very low, the OptiBIRTH intervention may be more effective within a relatively shorter time frame. The VBAC rate in control sites in Italy also rose from 10**.**6 to 21**.**3% in the same time, which may be due to the Hawthorne effect where being involved in the study, even in the control group, may have encouraged clinicians to review their practice, and offer VBAC rather than elective repeat CS. In addition, the control sites were offered the intervention in 2016, once the trial was complete, which may have assisted them to change.

Secondary outcomes were compared using the birthing data available for women who agreed to join the trial (intervention: 1174; control: 782). There was a statistically significant absolute increase of 4**.**3% in the VBAC rate (95% CI: 0 to 9%) (32**.**8% in the intervention group, 28**.**5% in control) (RR: 1**.**16; 95% CI: 1**.**01 to 1**.**33; *p* = 0**.**03). However, when this was adjusted for baseline characteristics, the difference was no longer statistically significant. No differences were seen in rates of induction or acceleration of labour between the two groups. This increase in VBAC among women who took part in the intervention may indicate that the intervention needs to be encouraged across the board, with both clinicians and women taking part to ensure any change in practice.

A break-down of the results shows apparent anomalies in some countries. For example, in the intervention sites in Ireland there was an overall drop in VBAC rates from 32**.**1% (2012) to 25**.**6% (2016), while the data from women participating in the trial in those sites showed a VBAC rate of 34**.**2%. This suggests that the VBAC rate for women not in the trial in 2016 was much lower than the 2012 norm. Evidence to support this comes from the drop in VBAC rates between 2012 and 2016 occurring, for some reason, in one of the three Irish intervention sites only. Recent work on VBAC rates in two of the largest maternity hospitals in Ireland corroborates this evidence, showing a steep decline from 64**.**4% in 1990 to 32**.**9% in 2014, with rates still falling from 2012 to 2014 [32]. Similar decreases were also shown in Massachusetts, from 32% to below 10% for the decade 2000 to 2010, and in one area of Germany, which showed a decrease from 48% in 1990 to around 25% in 2012 [32]. These decreases may be a reaction to the publication of a number of studies in the late 1990s and early 2000s that highlighted the risks of VBAC, without taking account of the international consensus that VBAC, using evidence-based guidelines, is a clinically safe choice for most women with a previous CS [32]. The decrease in VBAC rates in the Irish intervention sites over the time-period of the OptiBIRTH study (2012 to 2016) may also have been affected by many adverse reports in the Irish media, including an investigation into maternity care practices at one of the participating sites and, in particular, one situation where a maternity hospital sought a court order (unsuccessfully) to compel a woman to have a CS [33]. Due to the use of a cluster randomised trial methodology, the intervention could not be changed or modified to suit either different countries or altered circumstances in one country during the study. Using an implementation science approach might have allowed researchers and clinicians to modify the educational intervention in response to changing conditions.

Elective CS differed between the two groups (42**.**9% in intervention and 50**.**3% in control), perhaps indicating that the intervention had some effect in supporting women and clinicians in deciding to plan a VBAC. However, there was an increase in the rate of unplanned CS prior to labour in the intervention group (12% compared to 7**.**3%), which may have indicated a change of mind, or the emergence of clinical factors preventing a planned VBAC.

As 75% of all women entering labour and planning a VBAC in this study, had a successful VBAC, the avoidance of elective CS is key in increasing VBAC rates [34]. In terms of safety, there were two full uterine ruptures in the main OptiBIRTH trial (one in each group), a rate of 1 per 1000, which is lower than the published rate of 2.1 per 1000 maternities previously identified [35]. Intra-uterine and neonatal death rates showed no difference between intervention and control groups, and were equivalent to or below the lowest quoted European rates [36]. Other neonatal outcomes examined (admission to NICU, and preterm birth) also did not differ significantly between the two groups. Separate economic analysis showed that VBAC resulted in a cost reduction ranging from €3,334,052 (Germany) to €66,162,379 (Ireland) and gains in quality-adjusted life-years from 6399 (Italy) to 7561 (Germany) per 100,000 women birthing in each country [37].

Limitations of the OptiBIRTH cluster trial include missing data from 46 of the 2002 recruited women who moved away, or gave birth in another maternity unit (2**.**3%). The heterogeneity seen in the various sites is also an unavoidable issue in cluster randomised trials, and is a reality in ‘real world’ research.

The results of this trial may not be generalisable, because other settings, with different participants, fewer or more resources, differing extents of training or motivation of healthcare professionals and more positive or negative media foci, could produce different results. In this study, in settings with high rates of repeat CS for women with one previous CS, clinicians and women alike may view repeat CS as usual and beneficial. However, our economic analysis showed that women who expressed a preference for a vaginal birth, but who gave birth by elective repeat caesarean section, had impaired health-related quality of life at 3 months postnatal [38]. Belief in increased rates of uterine rupture with planned VBAC, and exaggerated fears of the ensuing complications [39] thus should not prevent clinicians from offering, and supporting women to achieve, VBAC within the context of genuine shared decision-making [40]. Providing the OptiBIRTH intervention in other settings could produce different results, especially if time is spent informing clinicians and policy-makers of these results, and the safety of the intervention, when applied as it was in this study. The qualitative aspects of this study showed clearly that a national culture that supports VBAC, and care in pregnancy and labour from confident and supportive clinicians are essential for success [41]. In particular, using an implementation research approach to introduce evidence-based interventions could be much more beneficial in that clinicians would be helped to identify and resolve existing barriers to supporting VBAC, and the intervention could be tailored to suit each site.

In summary, our results showed no significant difference in adverse maternal or neonatal outcomes between women exposed to the OptiBIRTH intervention and those who were not. There was no statistically significant difference in the change in the proportion of women having a VBAC between intervention and control sites. At individual site level, however, the results appear to show that the OptiBIRTH intervention may assist in supporting VBAC in sites with very low VBAC rates. As elective repeat CS is the most common reason for CS in multiparous women, and contributes to at least 10% of all CSs [42], interventions that are feasible and safe, and that have been shown to lead to a decrease of elective CS should be promoted. Continued research to refine the best way of promoting VBAC is essential, and this may best be done using an implementation science approach that can modify evidence-based interventions in response to changing clinical circumstances.

## Conclusion

This cluster randomised trial tested the effectiveness of the OptiBIRTH intervention in 15 sites in three countries and found no statistically significant difference in the change in the proportion of women having a VBAC between the intervention sites and the control sites. Importantly, and in terms of safety, there no differences in rates of uterine rupture, intra-uterine and neonatal death rates between intervention and control groups. The proportion of women having a VBAC in intervention sites with the lowest VBAC rates at the start of the trial rose by over 11% in the year after the trial concluded. This may indicate that the OptiBIRTH intervention is more effective in hospitals or countries with very low VBAC rates. CSs rates increased and VBAC rates decreased over time so it should not be surprising that it takes time to reverse this trend. Our concluding message is that the OptiBIRTH intervention is feasible and safe. The key components include an educational intervention provided by an opinion leader, evidence-based, unbiased information on the benefits and limitations of VBAC for women and, importantly, the option to become genuine, informed decision-makers in their care.

Continued research to refine the best way of promoting VBAC is essential. This may best be done using an implementation science approach that can modify evidence-based interventions, such as OptiBIRTH, in response to changing clinical circumstances.

## Supplementary information

**Additional file 1.** List of Research Ethics Committees and letters of approval.

**Additional file 2.** BMI at recruitment.

**Additional file 3.** Gestational age at recruitment.

**Additional file 4.** Maternal age at recruitment.

## Data Availability

The dataset used and analysed during the current study are available from the corresponding author on reasonable request.

## Abbreviations

BMI: Body mass index
CI: Confidence interval
DMC: Data monitoring committee
ICC: Intra-class correlation coefficient
ISRCTN: International standard randomised controlled trial number
NICU: Neonatal intensive care unit
OR: Odds ratio
RR: Risk ratio
TSC: Trial steering committee
VBAC: Vaginal birth after caesarean section
