# Supplementary material for: OptiBIRTH: a cluster randomised trial of a complex intervention to increase vaginal birth after caesarean section
Source: BMC Pregnancy Childbirth. 2020 Mar 6;20:143. doi: 10.1186/s12884-020-2829-y (PMC7059398; doi:10.1186/s12884-020-2829-y)
Supplement: Supplementary file 1 — Additional file 1. List of Research Ethics Committees and letters of approval. [file 12884_2020_2829_MOESM1_ESM.docx]

| **Country** | **Name of Committee** |
| --- | --- |
| *Finland* | University of Eastern Finland Committee on Research Ethics |
| *Sweden* | Regionala Etikprövningsnämnden i Göteborg, 413 20 Göteborg |
| *Netherlands* | Medisch Ethische Toetsingscommissie (METC - Atruum-Orbis-Zuyd) |
| *Germany* | Ethik-Kommission der Landesärztekammer Hessen, 60488 Frankfurt |
| *Germany* | Ethikkommission der Medizinischen Hochschule Hannover, Carl-Neuberg-Str. 1, 30625 Hannover |
| *Germany* | Klinischen Ethikkomitee der Hospitalvereinigung St. Marien GmBH, 42109 Wuppertal |
| *Germany* | Geschäftsstelle der Ethik-Kommission an der Medizinischen Fakultät der Universität Leipzig, Haus: Karl-Sudhoff-Institut für Geschichte der Medizin und der Naturwissenschaften, 04109 Leipzig |
| *Italy* | Comitato Ethico Indipendente dell'Azienda Ospedaliero-Universitaria di Bologna, Policlinico S. Orsola-Malpighi , Via Albertoni, 15 - 40138 Bologna |
| *Italy* | Comitato Ethico dell'Azienda Ospedaliera Universitaria della Seconda Università degli Studi di Napoli, Italy |
| *Italy* | Comitato Etico dell'Azienda Ospedaliera Universitaria "San Martino" di Genova - Largo Rossana Benzi,10 - 16132 Genova |
| *Italy* | Comitato Etico Indipendente Locale dell'Azienda Ospedaliera Ospedale Policlinico Consorziale di Bari - Piazza Giulio Cesare,11 - 70124 Bari |
| *Italy* | Comitato Ethico Servizio Sanitario Nazionale Regione Plemonte O.IR.M/S.Anna- Ordine Mauriciano Di Torino - 10125 Torino |
| *Ireland* | Faculty of Health Sciences Research Ethics Committee, Trinity College Dublin |
| *Ireland* | Ethics Research Committee Health Service Executive (HSE) West (MidWestern Regional Hospital, Limerick) |
| *Ireland* | Clinical Research Ethics Committee, HSE - Galway (Galway Regional Hospitals Research Ethics Committee) |
| *Ireland* | Research Ethics Committee HSE-Midland Area - Tullamore, Co. Offaly |
| *Ireland* | Healthcare Research Advisory Committee (HRAC) (HSE Dublin North East) |
| *Ireland* | Clinical Research Ethics Committee of The Cork Teaching Hospitals (UCC) |

**Additional file: List of Research Ethics Committees who granted approval**
