# Supplementary material for: OptiBIRTH: a cluster randomised trial of a complex intervention to increase vaginal birth after caesarean section
Source: BMC Pregnancy Childbirth. 2020 Mar 6;20:143. doi: 10.1186/s12884-020-2829-y (PMC7059398; doi:10.1186/s12884-020-2829-y)
Supplement: Supplementary file 2 — Additional file 2. BMI at recruitment. [file 12884_2020_2829_MOESM2_ESM.docx]

**Additional file 2 BMI at recruitment**

| **Country** | **Intervention** | | | **Control** | | |
| --- | --- | --- | --- | --- | --- | --- |
|  | **<25** | **25.00-29.99** | **≥30** | **<25** | **25.00-29.99** | **≥30** |
| **Trial as a whole** | 557 | 350 | 202 | 310 | 268 | 176 |
| **Germany** | 233 | 130 | 78 | 126 | 89 | 54 |
| **Ireland** | 152 | 130 | 80 | 107 | 81 | 60 |
| **Italy** | 172 | 90 | 44 | 77 | 98 | 62 |
