# Supplementary material for: OptiBIRTH: a cluster randomised trial of a complex intervention to increase vaginal birth after caesarean section
Source: BMC Pregnancy Childbirth. 2020 Mar 6;20:143. doi: 10.1186/s12884-020-2829-y (PMC7059398; doi:10.1186/s12884-020-2829-y)
Supplement: Supplementary file 3 — Additional file 3. Gestational age at recruitment. [file 12884_2020_2829_MOESM3_ESM.docx]

**Additional file 3 Gestational age at recruitment**

| **Country** | **Intervention** | | | **Control** | | |
| --- | --- | --- | --- | --- | --- | --- |
|  | **<30 weeks** | **30-32 weeks** | **≥33 weeks** | **<30 weeks** | **30-32 weeks** | **≥33 weeks** |
| **Trial as a whole** | 647 | 104 | 442 | 483 | 46 | 276 |
| **Germany** | 112 | 57 | 297 | 107 | 24 | 157 |
| **Ireland** | 324 | 16 | 26 | 244 | 2 | 8 |
| **Italy** | 211 | 31 | 119 | 132 | 20 | 111 |
