# Supplementary material for: OptiBIRTH: a cluster randomised trial of a complex intervention to increase vaginal birth after caesarean section
Source: BMC Pregnancy Childbirth. 2020 Mar 6;20:143. doi: 10.1186/s12884-020-2829-y (PMC7059398; doi:10.1186/s12884-020-2829-y)
Supplement: Supplementary file 4 — Additional file 4. Maternal age at recruitment. [file 12884_2020_2829_MOESM4_ESM.docx]

**Additional file 4 Maternal age at recruitment**

| **Country** | **Intervention** | | **Control** | |
| --- | --- | --- | --- | --- |
|  | **<40 years** | **≥40 years** | **<40 years** | **≥40 years** |
| **Trial as a whole** | 1099 | 96 | 728 | 75 |
| **Germany** | 427 | 39 | 265 | 24 |
| **Ireland** | 358 | 10 | 242 | 12 |
| **Italy** | 314 | 47 | 221 | 39 |
